# Electronic health information exchange in underserved settings: examining initiatives in small physician practices & community health centers

**DOI:** 10.1186/1472-6963-14-415

**Published:** 2014-09-21

**Authors:** J Mac McCullough, Frederick J Zimmerman, Douglas S Bell, Hector P Rodriguez

**Affiliations:** School for the Science of Health Care Delivery, College of Health Solutions, Arizona State University, Phoenix, AZ USA; Department of Health Policy & Management, Fielding School of Public Health, University of California, Los Angeles, USA; David Geffen School of Medicine, University of California, Los Angeles, USA; Division of Health Policy and Management, School of Public Health, University of California, Berkeley, USA

## Abstract

**Background:**

Health information exchange (HIE) is an important tool for improving efficiency and quality and is required for providers to meet Meaningful Use certification from the United States Centers for Medicare and Medicaid Services. However widespread adoption and use of HIE has been difficult to achieve, especially in settings such as smaller-sized physician practices and federally qualified health centers (FQHCs). We assess electronic data exchange activities and identify barriers and benefits to HIE participation in two underserved settings.

**Methods:**

We conducted key-informant interviews with stakeholders at physician practices and health centers. Interviews were recorded, transcribed, and then coded in two waves: first using an open-coding approach and second using selective coding to identify themes that emerged across interviews, including barriers and facilitators to HIE adoption and use.

**Results:**

We interviewed 24 providers, administrators and office staff from 16 locations in two states. They identified barriers to HIE use at three levels—regional (e.g., lack of area-level exchanges; partner organizations), inter-organizational (e.g., strong relationships with exchange partners; achieving a critical mass of users), and intra-organizational (e.g., type of electronic medical record used; integration into organization’s workflow). A major perceived benefit of HIE use was the improved care-coordination clinicians could provide to patients as a direct result of the HIE information. Utilization and perceived benefit of the exchange systems differed based on several practice- and clinic-level factors.

**Conclusions:**

The adoption and use of HIE in underserved settings appears to be impeded by regional, inter-organizational, and intra-organizational factors and facilitated by perceived benefits largely at the intra-organizational level. Stakeholders should consider factors both internal and external to their organization, focusing efforts in changing modifiable factors and tailoring HIE efforts based on all three categories of factors. Collective action between organizations may be needed to address inter-organizational and regional barriers. In the interest of facilitating HIE adoption and use, the impact of interventions at various levels on improving the use of electronic health data exchange should be tested.

**Electronic supplementary material:**

The online version of this article (doi:10.1186/1472-6963-14-415) contains supplementary material, which is available to authorized users.

## Background

Health information technology has the potential to improve the quality and safety of health care while reducing costs [1, 2]. Yet absent the ability to exchange data within and across organizations, these gains are likely to remain elusive [3]. At its core, health information exchange (HIE) entails the ability for multiple care providers and stakeholders to appropriately, efficiently, and securely access a patient’s medical information [4].

Electronic HIE initiatives have been undertaken across numerous health systems in a range of nations. For example, in Denmark uniformly high levels of IT utilization and a national health care system have enabled robust exchange of patient data through a national network [5]. In the UK, a recent national effort developed summary care records that are sharable across a secure network, though mixed results have been reported [6]. In New Zealand, a strong system has emerged that gives many providers the flexibility to exchange data with relevant organizational partners [7]. By most measures, the U.S. continues to lag many developed countries in electronic HIE, [7] perhaps due to the complex organization of providers, payers, and care delivery settings in the U.S. health care system [3].

In an effort to bolster America’s health information technology and HIE capabilities, the 2009 Health Information Technology for Economic and Clinical Health (HI-TECH) Act made available nearly $30 billion to encourage hospitals and clinicians in the United States to make “Meaningful Use” of information technology [8]. HIE is also important for the Accountable Care Organization and Patient-Centered Medical Home models that stem from the 2010 Affordable Care Act [9, 10]. To qualify for incentive payments, providers must, among other things, be able to exchange patient healthcare information electronically between providers and across clinics, [11] a capacity that many believe will help address both cost and quality concerns [1, 12–15]. Reductions in costs and improvements in quality have been reported in some but not all settings [16–18]. Variation in observed cost and quality outcomes may be at least partially reflective of the heterogeneity of HIE systems, data elements exchanged, care settings in which HIEs operate, and national health system features [7, 14, 19–22].

Currently, use of electronic HIE in the U.S. lags, with approximately 30% or less of U.S. hospitals and 10% of ambulatory practices having an HIE in place by 2012 [23]. This represents an increase from approximately 20% of hospitals as of 2009 [24]. Many HIEs reported difficulties in sustaining HIEs despite HI-TECH incentive payments currently available [23]. As a result, some HIE efforts are underperforming or failing altogether [11, 25].

Perhaps more concerning than low overall HIE use is evidence of differential adoption and use of HIE across provider types and care settings [26, 27]. Involvement of small- and medium-sized ambulatory practices has lagged relative to hospitals and large ambulatory settings [16, 28]. This disparity is especially important given that small ambulatory settings serve a disproportionate number of traditionally-underserved individuals [29]. Other sources of care for the underserved such as Federally Qualified Health Centers (FQHCs) face similar problems [30, 31]. For example, FQHC adoption of electronic medical records (a necessary precursor for HIE) lagged considerably relative to larger practices and hospitals [29, 30]. FQHCs and small physician practices are both critical components of America’s healthcare safety net, providing primary health care for millions in high-need communities [32]. These two settings are prime examples of providers ideally positioned to contribute to, and benefit from, electronic access to a client’s complete health record [20, 33, 34].

However, the cause of this disparity, and what might be done about it, is not well understood. For example very little data are available on barriers to HIE use by FQHCs. A study of nine organizations in the state of Colorado identified several hypothetically important HIE functions and means of facilitating adoption in small physician practices, including technical support and financial assistance [28]. A second study of nine primary-care practices in the state of Minnesota identified leadership and financial support as important facilitators of HIE adoption and use by small practices [35]. However both of these studies were conducted prior to the availability of HI-TECH Act incentives for HIE use.

Yet even after the introduction of Meaningful Use financial incentives for electronic exchange of health data, HIE usage remains low among small practices and community health centers [36]. This suggests that addressing financial barriers alone may be insufficient to reduce the disparity in HIE use between large hospitals and underserved settings such as small practices and FQHCs. What barriers remain unaddressed? What strategies might FQHCs or small physician practices consider to attempt to overcome these challenges?

Our study examines why adoption of HIE in small clinics remains relatively slow, with an ultimate goal of better understanding what might be done about it. This purpose of our study is to generate knowledge about the spread of HIE in underserved settings by examining barriers and benefits to the spread of HIE in smaller-sized primary care practices and FQHCs.

## Methods

We conducted primary data collection in two settings. Citrus Valley Health Partners (CVHP) is a provider network in the San Gabriel Valley, California. The network provides care for many traditionally underserved individuals, predominantly Hispanic, with over 40% of care going to underinsured and uninsured individuals. CVHP providers operate independently at small or solo practices and are free to select health IT suitable to their practice. The CVHP administration provides technical support and oversees centralized initiatives such as the *Collaborate* system, a web-based tool enabling all providers to view data exchanged from other CVHP sources (including three hospitals, an anticipated 90 providers, and laboratories in the community) and to securely message other providers. Data include care summaries, laboratory results, and hospitalization records and are available to be viewed by all participating providers at CVHP, regardless of whether a physician is contributing data to the system.

We also partnered with the Federally Qualified Health Center Urban Health Network (FUHN), a consortium of ten FQHCs in Minneapolis-St. Paul metropolitan area. The group recently partnered with the Minnesota Department of Human Services to operate an Accountable Care Organization for Medicaid patients. FUHN is governed by the CEOs or executive directors of each of the clinics and shares an administrative service contract. The consortium has begun implementing patient data exchange through an application known as *CentraHealth*, aimed at enabling electronic exchange between FQHCs and the hospitals serving their Accountable Care Organization (ACO) patients [34]. The system is still in its early stages but is planned to involve exchange of visit summaries and discharge records from hospitals to FQHCs. Bi-directional exchange of data back to hospitals would also be feasible.

### Conceptual framework

**Figure 1:**
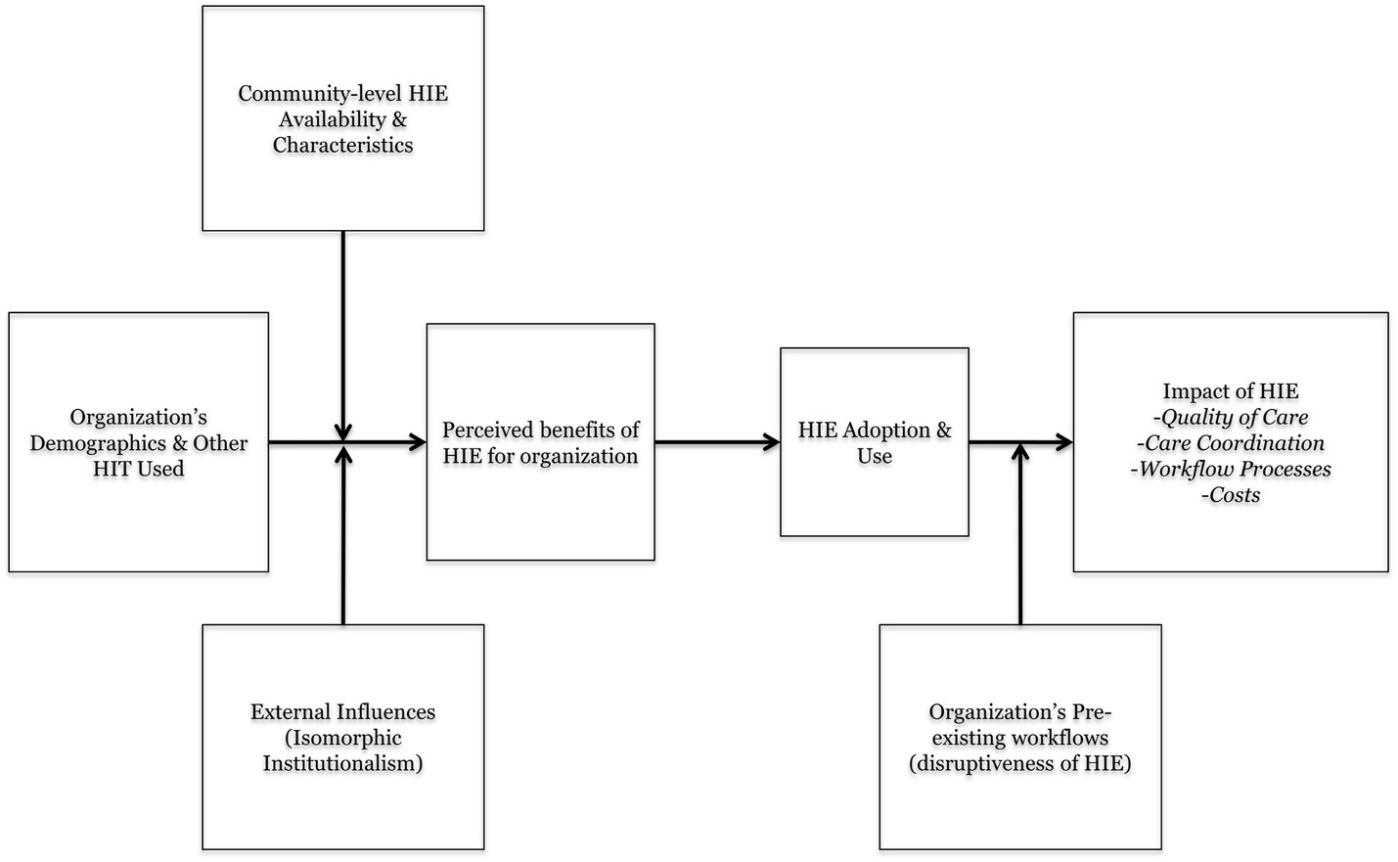
**Logic model of barriers and facilitators to HIE adoption within target population.**

We developed a logic model to shape development of our data gathering activities (Figure 1). We hypothesized that the availability and characteristics of existing HIE efforts (e.g., system-type, breadth and depth of information exchange), organizational demographics and patients served (e.g., number and specialty of physicians, type of practice, patient insurance or language spoken), and external influences (e.g., partner organizations or professional groups and the tendency for institutions to resemble their close or aspirational peers [37]) would impact a practice’s or clinic’s perceived utility of HIE. The perceived utility, in turn, would influence HIE adoption and use, as emphasized in the technology acceptance model [38]. Subsequent use of the system may then lead to an impact in terms of quality, care coordination, or costs. Impacts may be moderated by the extent that the HIE use disrupts pre-existing organizational workflows.

### Data collection

As we sought to gather information on the experiences, perceptions, and practices in these two care settings, study data was collected through semi-structured key-informant interviews [39, 40]. We developed interview guides with physician practices and with FQHCs, both of which were informed by organizational behavior and socio-technical theories [41, 42]. An iterative review and comment process was used to pilot-test the interview guides through initial interviews of physicians and administrators at both CVHP and FUHN. In the spirit of a partnered research approach, we modified the interview guides based on pilot-test input to cover topics of interest and benefit to both partner organizations. Interview guides are available in a supplementary online material (Additional file 1).

### Recruitment of study participants

For both community partners, study participants were purposefully selected to elicit a diverse range of experiences and opinions. We sought to interview individuals who would be involved in adoption decisions and integration of HIE into workflows at each organization, so we targeted physicians at the small-sized practices and administrators at FUHN clinics.

At smaller-sized practices, we targeted providers who were more- and less-frequently using the system to elicit ideas and opinions from both groups, based on usage-level input received from CVHP administration. We contacted all four of the practices involved in pilot-testing the *Collaborate* system and enrolled three of those practices. We contacted the 20 practices that were not involved in *Collaborate* pilot-testing, including those with relatively lower levels of system use as of September 2013, and enrolled eight of those practices. At FUHN, we contacted and successfully enrolled five FQHCs (of the ten total clinics in FUHN), interviewing a range of administrators and IT professionals. All recruitment was conducted via email using contact information provided by CVHP or FUHN staff or via telephone using contact information available online.

### Interview data

We conducted interviews with a total of 24 providers, administrators, and office staff in 16 practices and clinics. 20 of the 24 interviews were conducted in person. Informed consent was granted by all participants; 22 of 24 interviewees agreed to have interviews digitally recorded. Field notes were taken for the remaining two and transcribed immediately following completion of the interviews. Four interviews were conducted via phone and were digitally recorded. Recordings were professionally transcribed and spot-checked for accuracy. Interviews lasted 20 – 60 minutes (median = 32 minutes), for a total of more than 12 hours of recorded interviews plus two sets of field notes from non-recorded interviews.

### Data analysis

Transcripts were initially coded using an open-ended approach, allowing theories to emerge from the data [43]. A second round of coding was then performed to identify themes from the open-coding approach and from our logic model. Similar approaches have been used in other qualitative studies of health IT or HIE [15, 28, 44]. Coding practices were examined and refined during regular research meetings. Coded transcripts were reviewed by a researcher not involved in individually coding the manuscripts to identify uncoded instances of HIE facilitators and barriers. This revealed few instances of relevant uncoded material. Transcripts were analyzed using Atlas.ti version 7.1. The study protocol was reviewed and approved by Institutional Review Boards and UCLA (#13-000353) and the University of Minnesota (1306S35201). Our research adheres to the RATSÂ© qualitative research review guidelines, which emphasize the relevance of the study question, appropriateness of qualitative methods, and transparency of procedures [45].

## Results

Summary characteristics of study practices and clinics are summarized in Table 1 (small-practices) and Table 2 (FQHCs). The FQHC’s *CentraHealth* system was in the implementation and adoption phase at the time of the interviews. Each practice was therefore able to offer views on the system as they saw it impacting their own work and ability to access information from external sources.

**Table 1:** **Summary characteristics for small-size practice interviews**

Practice site	Practice specialty	Interviewee	Provider characteristics		Practice characteristics			
			Multi-lingual	Years in practice	# Physicians	# administrative FTEs	Transitioned from paper to EMR	Involved in***Collaborate***pilot
A	Family Medicine	• Physician	Yes	> 30	1	3	No	Yes
		• Office Manager						
B	Obstetrics	Physician	Yes	10 – 20	1	2	Yes	Yes
C	Family Medicine	• Physician	Yes	< 10	2	3	Yes	No
		• Physician	Yes	< 10				
D	General Surgery	Physician	No	> 30	1	2	Yes	No
E	Internal Medicine	• Physician	Yes	> 30	1	3	No	No
		• Office Manager						
		• Front Office Clerk						
F	Internal Medicine	Office Manager	No	20 – 30	2	2	No	Yes
G	Family Medicine	Physician	No	20 – 30	1	2	Yes	No
H	Pediatrics	Physician	Yes	< 10	1	3	Yes	No
I	Family Medicine	Physician	No	10 - 20	1	2	Yes	No
J	Pediatrics	• Physician	Yes	10 – 20	3	4	Yes	No
		• Office Manager						
K	Internal Medicine	Physician	No	> 30	1	2	No	No

**Table 2:** **Summary characteristics for FQHC interviews**

Clinic site	Interviewee(s)	Annual visits	Using Epic EMR
1	• Director	> 30,000	No
	• Information Manager		
2	Director	> 30,000	Yes
3	• Executive Director	10,000 – 20,000	No
	• Information Manager		
4	• Director	10,000 – 20,000	Yes
	• Administrative Manager		
5	Director	20,000 – 30,000	No

### Aggregated barriers & benefits

We aggregated the barriers to and benefits of HIE as identified during interviews with CVHP practices and FUHN clinics. We present these in Table 3 and Table 4 respectively along with accompanying exemplar quotations. Our analysis revealed that the barriers and benefits of HIE use in both CVHP practices and FUHN clinics tended to concentrate according to three levels within the healthcare system: intra-organizational, inter-organizational, and regional (Table 5). It was apparent that the barriers to HIE in these two settings were most salient at the inter-organizational and regional levels. However, the benefits to HIE did not accrue to the same level or breadth of stakeholders. Specifically, the benefits we identified were most salient at the intra-organizational level and pertained largely to the patient and or the provider. Findings are presented below grouped according to whether they represent a barrier to HIE or a benefit of HIE.

**Table 3:** **Barriers to HIE for small physician practices and FQHCs, with exemplar quotes**

Type of barrier	Example quote from practice/clinic
Lack of well-functioning area-level exchange	“I think if we did have communication with more entities it would be better. I think in certain parts of the country… they’re all integrated. They know exactly what happened to the patient ten years ago in all of their records because they use the same system. But now if there’s thousands of EMR companies there’s a lot of integration issues and there’s a lot of red tape. It’s hard to actually to communicate with other people outside of our sister”.
Market characteristics, including number, type, and size of partner organizations	“The reality is these systems are very expensive. They’re not easy to manage, overall, and sometimes the smaller clinics, as you’re probably hearing from the primary care clinics, you don’t always have the internal sophistication to go ahead and support them to the level and that’s where we struggle”.
Relationships or previous experiences with exchange partners	“It just comes down to priorities. We’re so far down the priority list for [the hospital organization] to even contemplate doing a direct interface with [us] that it’s time commitment prohibitive, and cost prohibitive for them”.
Challenge achieving a critical mass of users	“I’m a surgical specialist, so I have to wait until there are enough primary care physicians who are online who may refer me a patient or who we may have a mutual patient. So from a practical point of view, I don’t use it that much because I’m still waiting to get that information”.
Health IT used (e.g., type of EMR used & integration into organization’s workflow)	“It seems like it’s designed really well and you’ve thought of everything but when it gets back there and you realize they are completely overwhelmed by all these additional things that they have to do at every visit, there is just not any more room to do anything at every visit.
	“The other concern was how efficient is it to have two systems right next to each other? Our doctors don’t have time to do that. Our MAs don’t have time to do that. So there were some logistical concerns that we were very hesitant about”.
Data ownership and provider liability concerns	“Unfortunately what we’re really finding out here in spades is that that [HIPAA] is in conflict with the efforts to manage care appropriately because it’s just had this chilling effect on being able to share information. [Providers] are not saying hold on to be obstructionists. There just saying hold on because if I give you that piece of information I have just committed a HIPAA violation”.

**Table 4:** **Benefits of HIE for small physician practices and FQHCs, with exemplar quotes**

Type of benefit	Example quote from practice/clinic
Improved productivity at initial visit	“When I get the information from the hospital or other providers, there is more value for the patient. I can know more even for the first visit. And usually can get more accomplished during that first visit than if I have to repeat all of the info that’s already in the system from somewhere else”.
Improved completeness of patient records	“I guess has a patient who was to the emergency room 84 times in a year. Eighty-four times in a year. I think [we] knew about five of them or something like that… For the first time our providers are seeing a chunk of clinical information about their patients that they’ve never had access to and that’s been a pretty revolutionary impact for them”.
Avoidance of duplicative services/patient financial risk	“During the initial visit, you can see if they had the labs done. You won’t duplicate any labs that were recently done and the patient wouldn’t have to pay out-of-pocket if you repeated those tests or x-rays. Also, it’s just better care. Let’s say you had a condition where you really needed to get that lab, I just think it’s better care”.
Improved non-visit consults	“I had a patient with lung cancer who called me at two in the morning because he was anxious. He was having shortness of breath. He couldn’t breathe correctly. I was able to actually use [HIE] data from his previous encounters in the hospital and his other providers. I saw what his actual oxygen saturation was, so I just told them maybe you better just go ahead and call 911. He actually ended up in the ICU”.

**Table 5:** **Levels of barriers and benefits to HIE in small physician practices and FQHCs**

Level	Types of barriers cited	Types of benefits cited
Regional	- Lack of well-functioning area-level exchange	
	- Large number/diverse range of partner organizations difficult to incorporate	
Inter-Organizational	- Heterogeneous relationships and previous experiences with exchange partners	
	- Challenge achieving a critical mass of users	
Intra-organizational: providers and/or patients	*Providers:*	*Providers:*
	- Lack of integration into organization’s pre-existing workflow	- Completeness of patient records
	- Data ownership and liability concerns	*Patients:*
		- Avoidance of duplicative services and financial risk
		- Improved productivity at initial-visit
		- Improved non-visit consults

### Benefits of HIE use

There was near unanimity in each CVHP practice’s and FQHC clinic’s expressed data-exchange needs—a more complete patient record. This was especially true for the patients each FQHC is responsible for under the FUHN ACO. Many FQHC-based interviewees shared an understanding of current challenges facing FQHCs with respect to external electronic exchange of data. Their market is without a regional health information exchange organization, so electronic exchange efforts are generally established on an as-needed basis between organizations.

Nearly all interviewees from CVHP practice sites expressed positive sentiments about the *Collaborate* system in the abstract. Timeliness of information was among the most frequently cited benefits, as was the attractive user-interface. One of the most frequently discussed ways in which the exchange impacted these CVHP practices was in terms of workflow. We found instances in which the system improved workflow. For example, one CVHP practice that had completed the transition from paper to electronic records noted an improvement in workflow: “[With *Collaborate*] *I can go to that one location, download it…I can even review before the patient even gets here and that saves time*”. However, we also found instances in which *Collaborate* appeared to hinder workflow. Practices A and E, neither of which had completed the transition from paper to electronic medical records, both noted added work from the system due to having to print out information separately for each patient through additional clicking, then manually adding the information to the patient charts. Neither indicated any sort of permanent re-working of processes as a result of *Collaborate*. No CVHP physicians reported making substantial overhauls of practice workflows as a result of adopting *Collaborate*. The four providers who had yet to complete the transition away from paper charts consistently reported difficulties integrating the information from the system into their patient visits.

While no CVHP physicians reported specific financial savings for their practices as a direct result of system usage, nearly all felt that there was some level of savings as a result of using the system:

*Right now there’s lots of discussion but just because you’re speaking to someone that feels like, yeah, this is a great benefit, he’s got to convey that to the higher ups who have to have that same sense. When it comes down to half a million dollars, or I have no idea I’m just throwing that number out, but to upgrade the equipment for the connectivity, where’s the return on investment? You can’t really quantify that for somebody.*

An area that was discussed by some as a barrier and others as a potential facilitator to HIE use was the high prevalence of Epic^®^ EMR use by hospitals and health systems in the Minneapolis-St. Paul market. The FQHC interview participants who did not have Epic or have plans to adopt it unanimously agreed that not having it was a substantial barrier to electronic data exchange: “*There is no direct interoperability with the hospital systems here in the [Minneapolis-St. Paul] metro area. If you want to play in that world, you have to be Epic. So if you’re outside the Epic bubble, you’re not able to exchange information*”. One FQHC that already used Epic specifically noted that they recently adopted Epic in large part because they wanted to be able to be compatible with systems at nearby hospitals. They noted that, while the acquisition and maintenance costs for the system were high, they believed having easier potential for tie-ins to electronic data exchange with other health care delivery organizations would make the investment worthwhile in the long-run. Another FQHC was in the process of changing to Epic from a non-Epic EMR system. An FQHC stakeholder emphasized that funds and effort currently geared towards implementing the *CentraHealth* data exchange should be redeployed towards transitioning FQHCs to the Epic EMR system.

The as-needed approach to patient data exchange was cited as a substantial barrier by FQHC stakeholders. All organizations reported serving high proportions of patients that receive care from a limited number of hospitals, though their share of any given hospital’s total patients was relatively low. Consequently, each FQHC had stronger incentives to establish data exchange linkages with a hospital than any given hospital did: *“It just comes down to priorities. We’re so far down the priority list for [the hospital organization] to even contemplate doing a direct interface with [our FQHC] that it’s time commitment prohibitive, and cost prohibitive for them”.*

The FQHC’s HIE solution (*CentraHealth*) was seen by some as the best way to leverage their collective bargaining power to break through the perceived insularity of Epic and get the data they want:

*That’s a potential game changer for us. It will allow us to say, okay, hospital X, Y, and Z, who are all Epic, you guys develop one interface for CentraHealth and it will push the information down to us. We’ll actually go ahead and help pay for that plug-in, and then you’re plugging into one interface instead of five interfaces. Things like that to make it easier by having one central data repository.*

One of the most commonly cited barriers to *Collaborate* use by CVHP office managers was incomplete patient information. Several noted that it was sometimes easier, or at least more reliable, to access this information via an existing hospital-based system enabling electronic access to hospital records:

*When clinic is especially busy, even if I had [both systems] already pulled up on two screens, I would just go where I was more confident I could find the patient. With the [hospital-based system] I can be closer to 100% confident that I’ll find the patient there. Even if it’s harder to use or the information isn’t quite as good. I just don’t want to risk not finding the patient*.

Interviews with CVHP physicians did not reveal similar barriers to retrieving complete information on patients. Instead, several physicians expressed a preference for *Collaborate*’s layout and ability to access patient information compared to their EMR:

*When I launch into my EMR I’m in one specific patient. When I launch into Collaborate I see my patient list so I can see everything that’s happened on a patient of mine within a certain timeframe and then, individually, launch from Collaborate into each patient to see what has changed, what’s the delta from the last visit?*

CVHP physicians did not note, even after direct prompting, that patients had particularly strong concerns about *Collaborate*. Where concerns did exist, physicians found them to be easily allayed by discussing or demonstrating the finite range of data available through the system. We did, however, note concerns from multiple CVHP physicians regarding data, ownership, and liability issues: “*I put in data on my patients but who else sees that data? What are my legal responsibilities regarding that data? I think that was probably my only reservation about [Collaborate]”.*

## Discussion

Our study extends what is known about HIE adoption in settings currently underrepresented in HIE efforts, such as small physician practices or FQHCs, and the conditions under which HIE efforts may or may not flourish. Our results are among the first work done in this setting in the wake of the changing financial incentives brought about by payment reform under the HI-TECH Act and the Affordable Care Act.

In both underserved settings examined in this study, physicians, office managers, and clinic administrators expressed strong support for improved ability to electronically exchange information and reliably access that information. However, substantial barriers to this desired capacity were consistently reported. We discuss our findings in three distinct levels—regional, inter-organizational, and intra-organizational.

First, at the regional level, the lack of community-level HIE availability drove physician practices and FQHCs to develop custom solutions to address their data needs. Neither group reported having sufficient leverage to gain access to all of the data they needed and came up with the best solution possible given local realities and available funds. Lack of market power is not unique to the practices or clinics included in our study and may represent a consistent barrier and potential cause for disparities in HIE use. As observed in FUHN clinics, however, new models for care delivery and provider reimbursement may alter the financial calculus and provide additional incentives to include small provider practices and FQHCs in HIEs. Examples from other nations suggest that health care system factors such as payment or integration models can greatly influence the use and impacts of information systems and HIE [7]. This may help to explain why studies of HIE in small physician practices conducted prior to the HI-TECH Act identified financial barriers as far more important than observed in this study.

Second, inter-organizational influences were relevant and important in both settings. The number, size, and type of clinical and data partners were all important factors in determining HIE adoption and use. In the smaller-sized practices, there was a “Catch-22” of not having a sufficient base of clinicians regularly accessing and checking the system and therefore not finding the system useful enough to use themselves. In FQHCs studied, close and pre-existing ties were the primary reasons why the exchange was developed in the first place. These inter-organizational barriers and facilitators helped shape both HIE systems. Others considering adoption or use of HIE should carefully consider how to facilitate the rapid development of a critical mass of data and users and whether champions exist for their system. In contrast to the regional factors discussed above, these inter-organizational influences on HIE adoption and use may be less dependent on a nation’s health care system or incentives. For example, the presence of high levels of multi-functional EMR systems has not always translated into high levels of electronic exchange; a recent survey of 10 nations found only one nation (New Zealand) where over half of physicians report the ability to electronically exchange patient summaries and test results [21]. In the U.S., rates of HIE across organizations are far lower than overall rates of HIE (including exchange within the organization) [36]. This underscores the difficulties of bridging multiple organizations in an HIE effort and the need for strong inter-organizational relationships. Where such relationships do not exist, it may be necessary to foster participation by larger actors such as network hospitals to legitimize HIE efforts [46].

Third, looking at factors within the practices and clinics, it was clear that use of other health IT was central to adoption and usage of HIE. The practice of printing out patient charts and HIE data before patient encounters limited the range of interaction with the HIE system, likely because this is a substantial workaround, [44] and limits the ability of the physician to make use of the system for open-ended data gathering purposes [13, 15]. This may limit the impact of HIE systems in other settings given that fewer clinicians are regularly accessing the system and able to communicate with one another. Coupling HIE adoption with workflow redesigns may help ensure that HIE implementation is synergistic with modifications of tasks, policies, or incentives [47]. At least one study conducted prior to the HI-TECH Act identified workflow issues as being of primary importance to small- and medium-sized practices’ adoption and use of HIE [28]. The persistent importance of workflow design both before and after the availability of financial incentives for HIE use suggests greater attention to workflow modification may be necessary for adopters and users of HIE. Indeed, workflow and other intra-organizational factors account for at least four of the eight dimensions of Sittig and Singh’s sociotechnical model for health IT [41].

As has been observed in other iterations of electronic exchanges, the hypothesized and realized benefits of electronic health information exchange accrued largely to the stakeholders most directly involved in delivery of patient care. Both providers and administrators underscored the benefits of HIE use for patients, care coordinators, and clinicians. In both cases, however, funding for the HIE came mainly from a single source (clinics or practices), underscoring the challenge of finding sustainable funding sources to support ongoing exchange efforts [22].

Perhaps the most urgent challenge in promoting use of HIE in these two underserved settings, is the lack of alignment in the levels of barriers and benefits. Specifically, barriers we noted exist at the regional and inter-organizational level while the benefits we noted were largely at the inter-organizational level. This pattern of perceived barriers and benefits results in a collective action problem where substantial cooperation is necessary to overcome barriers to HIE. One potential avenue forward may be for smaller practices and clinics to partner with one another to address these asymmetries. Many current models for the acceptance and use of health IT do not explicitly account for the regional or inter-organizational barriers we identified, focusing instead on factors antecedent to an individual users’ decision to actually use a technology [38, 48]. Revised models that incorporate upstream influences may be well-suited to underserved settings.

Our study has some limitations to note. We collected data from providers and clinics actively involved in the adoption or use of the exchange efforts examined. The participant sample may differ from smaller-sized practices or FQHCs who do not adopt electronic data exchanges and our findings may not generalizable to these settings. Our sample was small, although it was purposefully selected. Interviews were conducted in two separate geographic locations (California and Minnesota); experiences in other areas may differ. The two HIEs studied were also in different lifecycle phases. As such, the barriers and benefits identified in each setting may be specific to the respective adoption and use phases when interviews were conducted.

## Conclusions

We found important facilitators and barriers to electronic data exchange in smaller-sized practices and FQHCs. In each setting, we examined an exchange initiative tailored to suit local needs and found evidence that adoption and use of the systems differed according to factors at three levels.

First, regional-level characteristics played an important part in determining the type of data exchange possible (e.g., through a regional HIE or not, number/size/type of partner organizations) and determining the practicality of exchanging data between organizations (e.g., in markets dominated by a given health system). Second, inter-organizational factors such as the presence of close and trust-filled relationships with exchange partners and achieving a critical mass of users. Third, intra-organizational factors such as the type(s) of health IT used within an organization (e.g., EMR brand or adaptation of workflow to fully leverage IT capabilities) was linked to whether and how the exchange system was used. These broad factors might facilitate the development of measures of the readiness of a region, a coalition, or an organization to participate in electronic data exchange, even in the absence of local federally-sponsored exchange programs, e.g., RHIOs.

Understanding the presence and interplay of multiple levels of HIE barriers and facilitators may help others in underserved settings to make a more informed and realistic assessment of how HIE may, or may not, work for them. Our study is the first to specifically delineate these challenges in this way. While we did not measure the relative importance of these factors, future work to confirm our hypotheses may be useful to practitioners attempting to better understand their readiness for HIE adoption and use. Given the varying mutability of some factors at these levels, future research should elucidate which factors are essential to address in an HIE implementation effort. While there may be limited solutions to overcome some of the barriers identified here, removing other barriers and taking advantage of facilitators may in fact be within the purview of practitioners and administrators seeking to adopt and use HIE.

## Electronic supplementary material

Additional file 1: **Supplementary Online Appendix: Interview Guides used for Semi-Structured Interviews with Small Physician Practices & FQHCs.** (DOCX 23 KB)
