# Supplementary material for: Electronic health information exchange in underserved settings: examining initiatives in small physician practices & community health centers
Source: BMC Health Serv Res. 2014 Sep 21;14:415. doi: 10.1186/1472-6963-14-415 (PMC4181433; doi:10.1186/1472-6963-14-415)
Supplement: Supplementary file 1 — Additional file 1: Supplementary Online Appendix: Interview Guides used for Semi-Structured Interviews with Small Physician Practices & FQHCs. (DOCX 23 KB) [file 12913_2014_3512_MOESM1_ESM.docx]

**Supplementary Online Appendix:**

**Interview Guides used for Semi-Structured Interviews with Small Physician Practices & FQHCs**

**Part A**

**Facilitators & Barriers to Use of HIE in Federally Qualified Health Centers**

**Key Informant Interview Guide**

1. **Current use of Health IT**

- Describe how your clinic currently records and stores patient records. Does your clinic use an electronic medical records system?
  - *If EMR = yes:*
    - What EMR system do you use?
    - How long have you had your current system?
    - Overall, how satisfied with the system are you?
    - What are your favorite features of the system?
    - What are your least favorite features?
  - *If EMR = no:*
    - Have you ever considered or investigated an EMR system for this clinic?
    - If so, what were some of the reasons you chose not to acquire an EMR at this clinic?
- Besides EMR, what are some of the major technological systems that your clinic uses or has plans to use?

1. **Current electronic exchange of clinical information**

Next, we will ask a few questions about your current access to and use of health information exchange, or “HIE”.

- Do you currently have any HIE underway at your clinic location?
- Have you had access to any type of HIE at previous places of employment?
  - If yes, please talk a bit about how that compares to what you currently have.
- [*If doesn’t have HIE*]:
  - Do you know of any existing HIE efforts in your clinic or in the community?
    - [*If yes*]: have you ever considered participating in it?
      - [*If yes*]: What factors played into your decision not to participate?

1. **Motivators, barriers, and incentives for adoption of HIE**

- [*If has HIE*]: *See questions at end of document. Not anticipating clinics to have HIE at this time, but questions have been prepared just in case*
- [*If doesn’t have HIE*]:
  - What do you think are some of the biggest barriers to you participating in a HIE?
    - (*Want to be sure to address barriers external and internal to the organization, if applicable*)
  - Have you identified or thought about any potential solutions to these barriers?
  - Hypothetically, if you could get an HIE set up:
    - what sort of benefits do you think participating in a HIE could offer your clinic, if any?
    - Hypothetically, what sort of drawbacks or downsides do you think there might be to HIE participation?

1. **CentraHealth Interface**

- Are you aware of the “CentraHealth” initiative?
- [*If no*]: *skip to next section*
- [*If yes*]:
  - What is your role in the project?
    - What is your organization’s role in the project?
  - What do you know about how the effort got started?
    - Whose idea was it?
    - Any major players or organizations that you feel were especially important to getting the ball rolling?
    - Were there other ideas for similar initiatives that were also considered?
    - How does this effort compare to previous efforts at information exchange for FQHCs in the metro area?
      - *(Especially interested in learning about concrete take-aways from previous efforts such as MNHIE*)
  - What sort of benefits do you anticipate seeing as the effort gets up and running?
    - Are there any anticipated drawbacks?
  - What are some of the biggest barriers you see to getting this up and running?
    - (*Want to be sure to address barriers external and internal to the organization, if applicable*)
    - Have you identified or thought about any potential solutions to these barriers?
  - In your opinion is this effort something unique to this Twin Cities-based FQHC and hospital network? Or is this something that could work in other cities or with other types of organizations?
    - Are there any keys to success that you’ve seen? Anything or anybody that was absolutely crucial to have?

1. **Community partnerships**

In order for HIE to be effective, it requires participation from other partners. Next we will discuss some of the partners in your community and how they are or might be integrated into the HIE efforts here.

- What types of organizations does your clinic interact with regularly?
  - [*Want to get enough info to be able to broadly categorize, don’t need names, contacts, etc.*]
- *[If HIE = no]* what organizations do you think would be important to involve in a future HIE?
  - Have you ever engaged or discussed potential HIE with these organizations?
    - [*If yes*]: Were both organizations receptive to adopting HIE? What were some of the issues that arose in those talks?

1. **Clinic decision-making**

Next, we have a few questions about your clinic’s decision-making structure.

- Can you please talk a bit about who is ultimately responsible for making decisions about acquisitions or technology upgrades?
- Who else is involved in these types of decisions? What is their role?

1. **Patient considerations**

Finally, we’d like to ask some brief questions about how you perceive HIE impacting the care your patients receive.

- Very briefly, can you talk about the patient population that you serve?
  - [*Want to get very general info—race/ethnicity, insurance, languages spoken, etc.*]
- What do you believe are the most major benefits to the patient that flow from your use of HIE?
- Do all of your patients benefit equally from HIE?
  - If not, what type(s) of patients benefit the most?
  - Are there any specific types of patients who you feel are not realizing the benefits?
    - (*E.g., language barriers, underserved or other vulnerable patient populations?*)
- What, if any, drawbacks do you anticipate for patients as a result of HIE?

1. **Wrap Up**

Those are all the questions I had prepared for today. Are there any other issues or topics that we did not discuss or areas you’d like to talk more about?

[*If no*]: Thank you very much for your time. We very much appreciate your contributions to this project.

***If Clinc has HIE, include these topics in the conversation:***

**Motivators, barriers, and incentives for adoption of HIE**

- Thinking specifically about your clinic, talk about the major reasons why you ultimately decided to participate in HIE.
  - What were some of the biggest motivating factors for initially deciding to participate?
    - Were there any specific benefits envisioned?
  - What were some of the biggest issues or sticking points that had to be worked out before you began participating?
    - Were there any specific problems envisioned?
  - Do you recall any specific incentives that were helpful in securing your participation?
    - Any particularly influential system features or functions?
    - Any particularly influential leaders, advocates, or system champions? (Can be either within or outside of the clinic)
- For your clinic today,
  - what are some of the biggest benefits you think HIE brings to your clinic?
  - what have been the most useful features?
    - Clinical documentation, Labs, Patient demographics, Referral management, (Others)?
  - are there any drawbacks to HIE in your clinic?

**Community Partnerships**

- What other organizations are involved in your current HIE?
  - Do they all participate equally?
  - Do you think that the data that each organization provides is equally helpful? Or are there organizations whose participation is more helpful than others?
- Are there other organizations that would be helpful to include in the information exchange? Why?
  - Have you had any trouble or push-back trying to get these organizations to participate in HIE?
  - Have you been able to overcome these issues with any partners? How?

**Part B:**

**Facilitators & Barriers to Use of HIE in Small-Sized Primary Care Practices**

**Key Informant Interview Guide**

1. **Conceptualization of HIE**

As you know, this interview will be about health information exchange. To begin the interview, what does “health information exchange” mean to you?

1. **Practice decision-making**

We will have a few questions about your practice’s decision-making structure.

- Can you please talk a bit about who is ultimately responsible for making decisions about:
  - how the practice is run on a day-to-day basis?
  - acquisitions or technology upgrades?
- Who else is involved in these types of decisions? What is their role?

1. **Current use of Health IT**

- Describe how your practice currently records and stores patient records. Does your practice use an electronic medical records system?
  - If EMR = yes:
    - What EMR system do you use?
    - How long have you had your current system?
    - Overall, how satisfied with the system are you?
    - What are your favorite features of the system?
    - What are your least favorite features?
  - If EMR = no:
    - Have you ever considered or investigated an EMR system for this practice?
    - If so, what were some of the reasons you chose not to acquire an EMR at this practice?
- Besides EMR, what are some of the major technological systems that your practice uses or has plans to use?

1. **Current electronic and non-electronic exchange of clinical information**

Next, we will ask a few questions about your current access to and use of HIE.

- Do you currently have access to Collaborate at this practice location?
- Have you had access to any type of HIE at previous places of employment?
  - If yes, how would you compare them with what you currently have?

1. **Motivators, barriers, and incentives for adoption of Collaborate**

- [*If has HIE*]: Thinking specifically about your practice, talk about the major reasons why you ultimately decided to participate in Collaborate.
  - If you were around at the time or have talked about it with others:
    - what were some of the biggest motivating factors for initially deciding to participate?
      - Were there any specific benefits envisioned?
    - what were some of the biggest issues or sticking points that had to be worked out before you began participating?
      - Were there any specific problems envisioned?
    - do you recall any specific incentives that were helpful in securing your participation?
      - Any particularly influential system features or functions?
      - Any particularly influential leaders, advocates, or system champions? (Can be either within or outside of the medical group)
  - For your practice today,
    - what are some of the biggest benefits you think Collaborate brings to your practice?
    - what have been the most useful features?
      - Clinical documentation
      - Labs
      - Patient demographics
      - Referral management
      - (One that doesn’t exist)
    - are there any drawbacks to HIE in your practice?
- [*If doesn’t have HIE*]:
  - Do you know of any existing HIE efforts at CVHP or in the community?
    - If so, have you ever considered participating in it?
    - [*If mentions Collaborate*]: Talk about the most major reasons why you do not participate in Collaborate.
  - Hypothetically, what sort of benefits do you think participating in a HIE could offer your practice, if any?
  - Hypothetically, what sort of drawbacks or downsides do you think there might be to HIE participation?

1. **Community partnerships**

In order for Collaborate to be effective, it requires participation from other partners. Next we will discuss some of the partners in your community and how they are or might be integrated into Collaborate.

- What types of organizations does your practice interact with regularly?
  - [*Want to get enough info to be able to broadly categorize, don’t need names, contacts, etc.*]
- Thinking specifically about Collaborate, what organizations would be the most helpful to include in the information exchange? Why?
- Have you had any trouble or push-back trying to get these organizations to participate in information exchange?
  - Have you been able to overcome these issues with any partners? How?

1. **Patient considerations**

Next, we’d like to ask some brief questions about how you perceive Collaborate impacting the care your patients receive.

- What do you believe are the most major benefits to the patient that flow from your use of Collaborate?
- Do all of your patients benefit equally from Collaborate?
  - If not, what type(s) of patients benefit the most?
- Are there any specific types of patients who you feel are not realizing the benefits?
  - Spanish or other non-English speakers?
  - Those without medical homes?
- Other vulnerable or underserved patient populations?

1. **Wrap Up**

Those are all the questions I had prepared for today. Are there any other issues or topics that we did not discuss or areas you’d like to talk more about?

[*If no*]: Thank you very much for your time. We very much appreciate your contributions to this project.
